# Patterns of knee osteoarthritis management in general practice: a retrospective cohort study using electronic health records

**DOI:** 10.1186/s12875-023-02198-z

**Published:** 2024-01-02

**Authors:** Ilgin G. Arslan, A. C. van Berkel, J. Damen, P. Bindels, M. de Wilde, S. M.A. Bierma-Zeinstra, D. Schiphof

**Affiliations:** 1 Department of General Practice, Erasmus MC University Medical Center, Rotterdam, The Netherlands; 2 Department of Orthopaedics, Erasmus MC, University Medical Center, Rotterdam, The Netherlands; 3 Department of Medical Informatics, Erasmus MC University Medical Center, Rotterdam, The Netherlands

**Keywords:** Knee osteoarthritis, Primary care, General practice, Electronic health records, Management patterns

## Abstract

**Objective:**

This study determined patterns of knee osteoarthritis (OA) management by general practitioners (GPs) using routine healthcare data from Dutch general practices from 2011 to 2019.

**Design:**

A retrospective cohort study was conducted using the Integrated Primary Care Information database between 2011 and 2019. Electronic health records (EHRs) of n = 750 randomly selected knee OA patients (with either codified or narrative diagnosis) were reviewed against eligibility criteria and n = 503 patients were included. Recorded information was extracted on GPs’ management from six months before to three years after diagnosis and patterns of management were analysed.

**Results:**

An X-ray referral was the most widely recorded management modality (63.2%). The next most widely recorded management modalities were a referral to secondary care (56.1%) and medication prescription or advice (48.3%). Records of recommendation of/referral to other primary care practitioners (e.g. physiotherapists) were found in only one third of the patients. Advice to lose weight was least common (1.2%). Records of medication prescriptions or recommendation of/referral to other primary care practitioners were found more frequently in patients with an X-ray referral compared to patients without, while records of secondary care referrals were found less frequently. Records of an X-ray referral were often found in narratively diagnosed knee OA patients before GPs recorded a code for knee OA in their EHR.

**Conclusion:**

These findings emphasize the importance of better implementing non-surgical management of knee OA in general practice and on initiatives for reducing the overuse of X-rays for diagnosing knee OA in general practice.

**Supplementary Information:**

The online version contains supplementary material available at 10.1186/s12875-023-02198-z.

## Introduction

Osteoarthritis (OA) is a highly prevalent progressive joint disease, causing pain and reduced function of the joint. It has been ranked as the 10th leading contributor to global disability [1–3]. Based on general practitioner (GP) registries, approximately 1.5 million people in the Netherlands were recorded with a code as having OA in 2019, mainly in the knee [4]. In a previous study [5], we developed algorithms to also identify knee OA diagnoses in the narrative data (i.e. free-text fields) of electronic health records (EHRs). Estimates using these algorithms were on average twice as high as estimates from codified data alone. This finding suggests that the current figures for the incidence and prevalence of knee OA from routine healthcare data, which are all based on codified data alone, are only half as high as the true figures.

As there is no cure for knee OA, management focuses on reducing symptoms and improving function. Current OA management guidelines [6–11] recommend core strategies of education and self-management, physical activity and exercise therapy, weight management, and walking aids as indicated, combined with oral or topical analgesics for pain reduction if necessary. Patients who do not benefit from these management modalities may opt for knee replacement surgery. Although current guidelines are consistent in their recommendations for the management of OA, evidence from several countries and healthcare settings show low adherence to guidelines in clinical practice [12–16]. Furthermore, patients often receive an X-ray referral for the diagnosis of knee OA even though it is not recommended in current guidelines. This can lead to unnecessary healthcare costs and can give the patient the wrong idea that OA is caused by ‘wear and tear’ [17, 18].

GPs are the first point of contact for patients and act as gatekeepers to secondary care (i.e. hospital care) in countries such as the Netherlands, the UK, and Scandinavian countries [19, 20]. They play a crucial role in the diagnosis and treatment of knee OA. The current guideline for GPs in the Netherlands exists since 2016. Whether the stepped care health model as described in the guideline is followed in the Netherlands is not yet known. Therefore, our main objective was to determine patterns in the management of knee OA by GPs in a real-world setting using routine healthcare data from Dutch general practices from 2011 to 2019. Previous research [21] showed that patients with less severe OA are less likely to have a codified OA diagnosis. This suggests that patients with severe knee OA are overrepresented in research that uses codified data alone. In contrast to previous studies, we incorporated both narrative and codified data from EHRs to select knee OA patients to examine knee OA management patterns in general practice across the entire spectrum of knee OA severity, thus providing a more accurate reflection of real-world clinical practice. In order to better understand the knowledge gap in previous studies that used codified data from EHRs alone, we additionally examined the differences in knee OA management between those with a codified diagnosis and those with a narrative diagnosis in their EHR.

## Method

### Design and setting

A retrospective cohort study was performed using the data of the Integrated Primary Care Information (IPCI) database. The IPCI database contains routine healthcare data from general practices covering approximately 2.5 million people in the Netherlands who are representative of the general Dutch population in terms of sex and age. A detailed description of the IPCI database has been given elsewhere [22–24]. In summary, the database contains structured and unstructured data from longitudinal clinical information on the medical journal in EHRs, documented using free-text notes by GPs, diagnoses according to the International Classification of Primary Care (ICPC) codes [25], laboratory findings, drug prescriptions, and referrals and correspondence between healthcare providers in primary and secondary care. EHRs from the IPCI database contain the majority of the patients’ medical information, as all citizens in the Netherlands are obliged to register with a GP, who acts as the first point of contact and the gatekeeper to secondary care [19, 20]. Data from the IPCI database were used from 1 to 2011 to 31 December 2019, as healthcare delivery was impacted by the first Covid-19 wave in March 2020 in the Netherlands.

### Study cohort

Dutch GPs are free to choose among competing information systems, which significantly differ in their features [23]. Data from GPs using an EHR information system with follow-up during the study period were used to select patients for the current study. Patients with knee OA were identified based on the ICPC code for knee OA (i.e. L90). In addition, patients with a knee OA diagnosis reported in narrative data (i.e. free text notes of healthcare providers) without an ICPC code for knee OA were selected to reduce the potential for selection bias. This method was developed in a previous study [5] and the algorithm to identify narratively diagnosed knee OA patients had a positive predictive value of 96%. The first diagnosis of knee OA was established as the index date, which could be based either on codified diagnoses (i.e. ICPC code L90) or narrative diagnosis (i.e. free text). Patients aged ≥ 30 with a first knee OA diagnosis (i.e. incident) between 1 and 2011 and 31 December 2016 and valid database history of at least 12 months prior to study entry were included in the study cohort. The entire database history available for the patient up to that index date were used to exclude patients with prior knee OA when identifying incident knee OA cases.

**Fig. 1:**
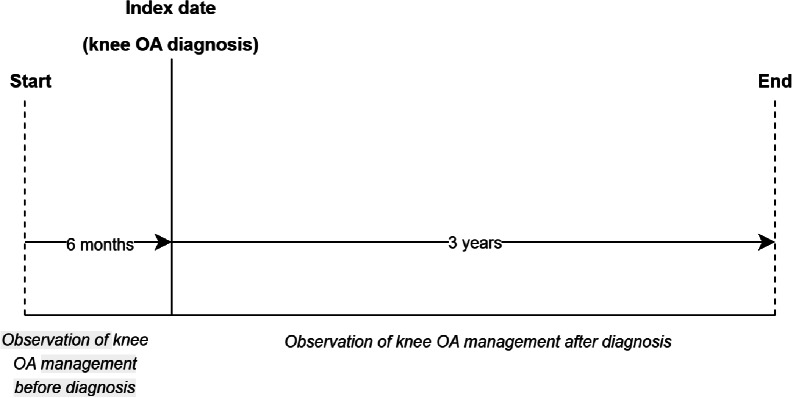
Details of the observation period of the included knee OA patients. *Note.* This figure shows details of the observation period of the included knee OA patients. Patients aged ≥ 30 with a first knee OA diagnosis (i.e. incident) between 1 July 2011 and 31 December 2016 and valid database history of at least 12 months prior to study entry were included in the study cohort. Patients were required to have at least three years of observation time after the index date, as pilot testing showed that most GP consultations for knee OA occurred within this period. Information was extracted from the full EHRs on the GP’s management at each consultation in this period from six months before the index date to three years after the index date

The full EHRs of 150 knee OA patients were assessed in a medical record review by one author (IGA, physiotherapist and researcher) to pilot test the patient selection. This showed that 10% of the patients were identified with knee OA in the GP information system based on a letter from an orthopaedic surgeon to the GP containing a summary of an orthopaedic consultation. In those cases, the GP’s referral to an orthopaedic surgeon occurred between three and six months before the index date. Therefore, an observation period of at least six months before the patient’s index date was required for inclusion (Fig. 1). In addition, patients were required to have at least three years of observation time after the index date, as pilot testing showed that most GP consultations for knee OA occurred within this period. Supplementary File 1 shows detailed information on the study design and patient selection.

Of the 184,492 knee OA patients identified with our algorithm in the IPCI database, a random sample of 750 was selected for the study cohort. The full EHRs of these patients were assessed against the eligibility criteria for the study by means of a medical record review by one author (IGA, physiotherapist and researcher), with scrutiny by a second author (JD, academic GP). Eligible patients were patients diagnosed with knee OA, meaning that the GP, healthcare provider in primary care (e.g. physiotherapist) or healthcare provider in secondary care (e.g. orthopaedic surgeon or radiologist) reported a knee OA diagnosis in the free text. Patients had to have at least one contact with their GP for knee complaints in the six months before or on the index date. Patients were excluded if they were diagnosed with knee OA in secondary care (report in specialist letter to the GP) only, since in such cases no management was provided by the GP. Patients were also excluded if they had a record of knee OA as an incidental finding on an X-ray or MRI after a traumatic event without any documentation of knee pain by the GP in the prior six months or immediately after a traumatic event, given the poor correlation between structural damage of the joint on imaging and the severity of symptoms [10, 26]. In addition, patients with a previous record of knee OA diagnosis or a total knee replacement in the medical history before the index date were excluded, since these patients had *prevalent* knee OA rather than *incident* knee OA. Patients with generalized OA (OA in more than one joint) or generalized pain without a specific management trajectory for knee OA were also excluded.

### Data collection

Patient demographics and characteristics were collected at index date, including sex, age, unilateral or bilateral knee complaints, and common comorbidities in patients with OA based on a systematic review [27]: (1) disorders related to metabolic syndrome; (2) heart/vascular diseases and events; (3) asthma; (4) Chronic Obstructive Pulmonary Disease (COPD); (5) spinal OA and hip OA; and (6) history of lower limb trauma, all based on ICPC codes (see Supplementary File 2 for the full list of ICPC codes).

Information was extracted from the full EHRs (including information documented in free text) on the GP’s management at each consultation in the period from six months before the index date to three years after the index date, up to a maximum of 15 consultations per patient. We divided the management information into the following categories based on current guidelines [6–10] and discussion within the research group: (1) wait-and-see/watchful waiting; (2) recommending a follow-up consultation; (3) education and self-management; (4) advice to lose weight; (5) advice on exercise/physical activity/sports; (6) advice to reduce physical activity/exercise or to take rest; (7) medication prescription or advice; (8) intra-articular injection; (9) aids and devices; (10) diagnostic work-up; 11) recommendation of/referral to other primary care practitioners; and 12) referral to secondary care. Supplementary File 3 presents detailed information on these categories. Patients could be treated according to more than one management category during one consultation. In patients who received a total knee replacement within three years after the index date, information was extracted up to the time of surgery.

### Statistical analysis

Descriptive statistics were used to describe baseline characteristics and the GP’s management from six months before to three years after the index date (knee OA diagnosis). Means and standard deviations (SDs), medians and interquartile ranges (IQRs), and counts (n) and percentages (%) were reported, as appropriate. Patterns of knee OA management for the first three consultations during the observation period were visualized using a Sankey diagram. A Sankey diagram shows nodes as actions taken by the GP during the consultation. The width of the flows between two nodes represents the numbers (i.e. proportion of all consultations with this management type) and pattern of the sequence from one action to another (e.g. medication prescription or advice followed by referral to secondary care). Differences in the GPs’ management modalities were assessed using the Chi-squared test with Yates’s continuity correction to compare: (1) patients with an X-ray referral during the observation period versus those without, (2) patients diagnosed with a codified diagnosis (ICPC code L90) versus those with a narrative diagnosis on the index date. Absolute differences in the percentage were reported, including the 95% confidence interval (CI). For patients who were narratively diagnosed on the index date and subsequently had a codified diagnosis, GPs’ management before and after the first codified diagnosis was described. The significance level throughout was set at two-tailed P < .05. Statistical analyses were performed using R Studio Software V.4.0.2.

## Results

### Characteristics of patients

**Fig. 2:**
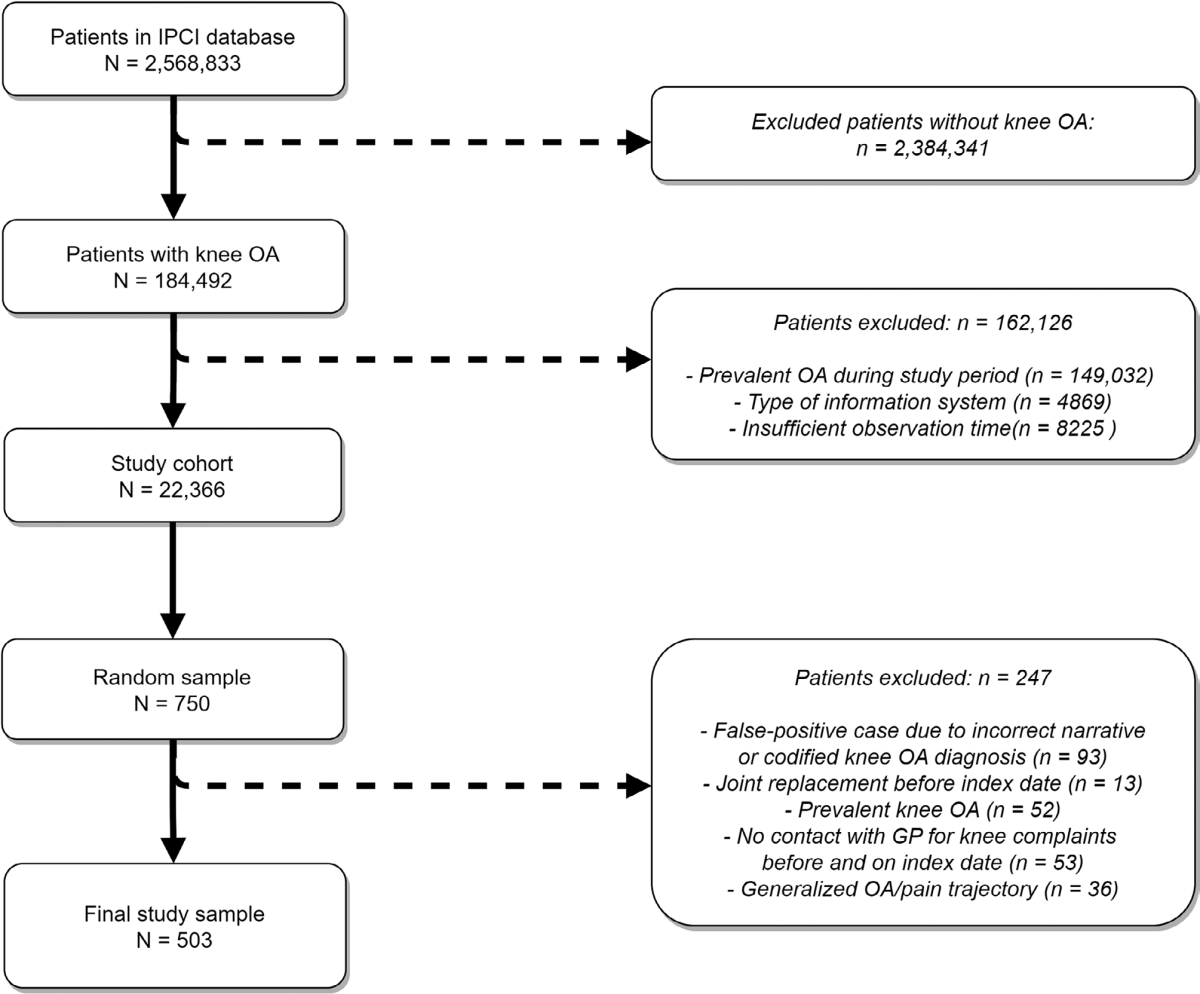
Flow chart of patients included in the study

A total of 503 patients met the inclusion criteria (Fig. 2). Of those, 40.0% (n = 203) were identified based on a codified diagnosis of knee OA (i.e. ICPC code L90) and 60.0% (n = 300) based on a narrative diagnosis on the index date (Table 1). Patients had a mean age of 64.3 (SD = 11.6) years, 60% (n = 302) were female and had unilateral knee complaints (82.5%, n = 415). Patients were diagnosed by a variety of healthcare providers during the total observation period; 71% (n = 357) by a GP, of whom 25.2% were only diagnosed by a GP and 74.8% by a GP and another healthcare provider. The majority of the remaining 29% (n = 146) patients were diagnosed by orthopaedic surgeons (41.1%), radiologists (28.8%), or orthopaedic surgeons and radiologists (15.8%).

**Table 1:** Patients’ characteristics

	Total (n = 503)
Age on index date, mean (SD)	64.3 (11.6)
Sex, female, n (%)	302 (60.0)
Type of record of diagnosis on index date, n (%) - Codified diagnosis - Narrative diagnosis * Received a code during the observation period after date of narrative diagnosis*	203 (40.4) 300 (59.6) 86 (28.7)
Diagnosed during observation period by, n (%) - GP * GP and other healthcare provider* * GP only* - Other healthcare provider (no GP) * Orthopaedic surgeon only* * Radiologist only* * Radiologist and orthopaedic surgeon* * Physiotherapist only* * Other combinations of abovementioned healthcare providers*	357 (71.0) 267 (74.8) 90 (25.2) 146 (29.0) 60 (41.1%) 42 (28.8%) 23 (15.8%) 3 (2.1%) 18 (12.3%)
Site of complaints during observation period, n (%) - unilateral - bilateral - unknown	415 (82.5) 67 (13.3) 15 (3.0)
Concurrent codified comorbidities, n (%) - Hyperlipidaemia - Hypertension - Diabetes - Myocardial infarct - Stroke/TIA - Peripheral arterial disease - COPD - Overweight - Asthma - Fibromyalgia - Rheumatoid arthritis - Hip OA - Spinal OA - Knee complaints code * Among patients with narrative diagnosis on index date* * Patients with codified diagnosis on index date* - Lower limb trauma	66 (13.1) 205 (40.8) 72 (14.3) 53 (10.5) 28 (5.6) 9 (1.8) 32 (6.4) 52 (10.3) 50 (9.9) 4 (0.8) 15 (3.0) 24 (4.8) 31 (6.2) 317 (63.0) *222 (74.0)* *95 (46.8)* 112 (22.3)

### Management recorded by the GP

**Fig. 3:**
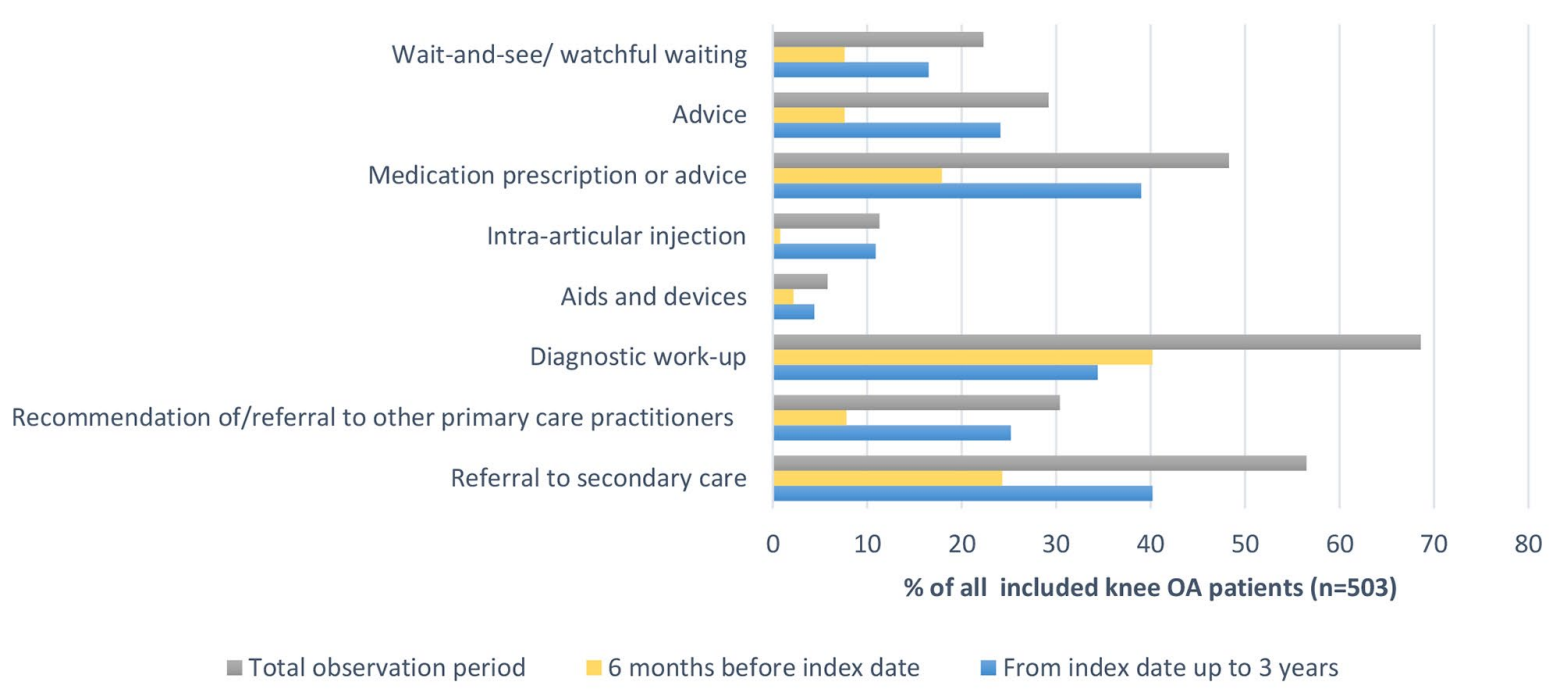
Management provided by the GP for knee OA patients during the total observation period, from 6 months before the index date to 3 years after the index date. *Note*: Percentages are based on the management on patient level. This means that patients were included as having a management modality when the modality was recorded at least once in the given observation period. The percentage is based on the total number of patients rather than the total number of management events. For example, during the total observation period (grey bar in Fig. 3) a referral of diagnostic work-up was recorded at least once in 68.6% of the patients. During 6 months before index date (yellow bar in Figs. 3), 40.2% of the patients had at least one record of X-ray referral and 34.4% of the patients at least one record after the index date (blue bar in Fig. 3)

Patients received a median number of three consultations (IQR = 3–4) for knee OA during the total observation period. The most widely recorded type of management by the GP was a referral of diagnostic work-up (68.6%), mainly in the form of an X-ray referral (Fig. 3, Supplementary File 4 for full details). Most patients (40.2%) received at least one X-ray referral before the index date and less frequently after the index date (34.4%). The second most widely recorded type of management by the GP was a referral to an orthopaedic surgeon (56.1%), most often after the index date. Medication was prescribed or advised in almost half of the patients (48.3%); mostly an oral NSAID and/or paracetamol. One third of patients (30.4%) were recommended of/referred to other primary care practitioners, mainly to a physiotherapist. Advice was recorded in one third of the patients (29.3%), mainly on education and self-management, and exercise/physical activity/sports. A recommendation of a follow-up consultation and advice to lose weight were recorded least often by the GP (0.8% and 1.2% respectively). Other less common types of management were: wait-and-see/watchful waiting policy (22.3%), corticosteroid injection (11.3%) and aids and devices (5.6%).

**Fig. 4:**
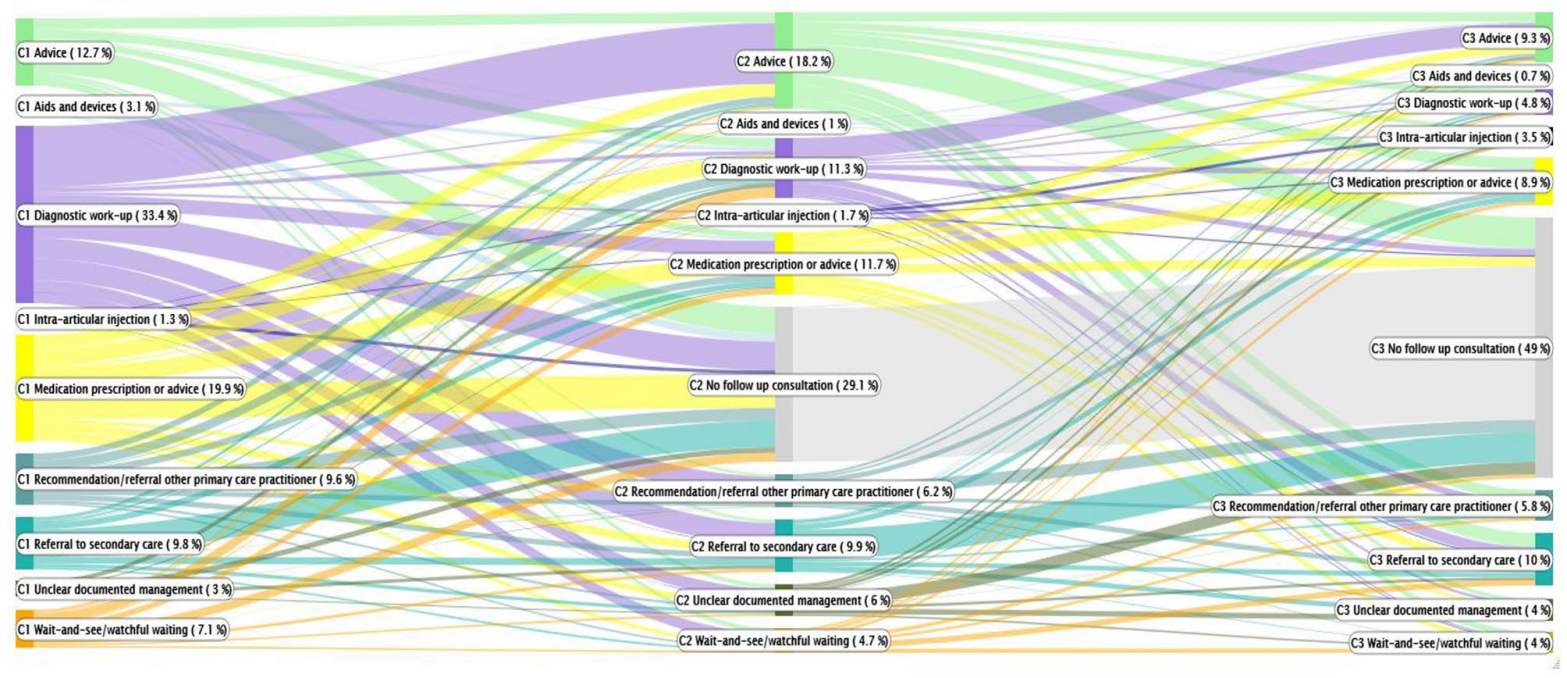
Sankey diagram visualizing the management of knee OA per consultation. *Note*: The Sankey diagram shows nodes as actions taken by the GP during the consultation. The width of the flows between two nodes represents the numbers (i.e. proportion of all consultations with this management type) and pattern of the sequence from one action to another (e.g. medication prescription or advice followed by referral to secondary care). The first node represents the management in the first consultation (N = 503 patients). The second node represents the management in the second consultation (N = 432) and the third node represents the management in the third consultation (N = 273). Percentages are based on the management in the first consultation. Patients could be treated using more than one management modality. The percentage is based on the total number of management events rather than the total number patients. For example, 33.4% of the patients received diagnostic work-up such as an X-ray on the first consultation (see first node in purple on the left). Of those, most patients received advice on the second consultation (see second node in green in the middle), followed by no consultation (see third node in grey on the right). See Supplementary Table 4 for more details of the management types

Figure 4 shows the recorded management of knee OA during the first three consultations. Patients could be treated using more than one management modality. The percentage presented in the Sankey diagram is based on the total number of management events rather than the total number patients. A referral for diagnostic work-up was the most widely recorded management modality during the first consultation (33.4%), followed by advice during the second consultation (18.2%). Medication prescription or advice was the second most recorded type of management during the first consultation (19.9%), followed by advice (12.7%) and referral to the secondary care (9.8%); in all three management modalities, it was most common for there to be no follow-up consultation. A referral to secondary care was recorded in 9.8% of the patients during the first consultation; again, there was then usually no follow-up consultation. A recommendation of/referral to other primary care practitioners during the first consultation was recorded in 9.6% of patients and was followed by a wide variety of management modalities during the second consultation. In most cases (49.0%) there were no further consultations after the second consultation, especially after a record of advice or referral to secondary care during the second consultation. In general, management recorded by the GP during the third consultation was very fragmented.

### Recorded management associated with X-ray referral

Medication prescription or advice and recommendation of/referrals to other primary care practitioners were recorded significantly more often in patients who received an X-ray (n = 318) compared to patients who did not receive an X-ray (n = 185) (absolute difference = 18.4% and 26.2% respectively) (Table 2). Patients who did not receive an X-ray were significantly more likely to be referred to secondary care compared to patients who received an X-ray (absolute difference = 19.4%). In patients who received an X-ray, all treatment modalities occurred more frequently after an X-ray, especially medication prescriptions and referrals to paramedics and secondary care (Supplementary File 5 for full details).

**Table 2:** Differences in management between patients who received an X-ray and patients who did not receive an X-ray during the total observation period

	Patients without X-ray referral (n = 185)	Patients with X-ray referral (n = 318)	Absolute difference (%) (95%CI) †
Total number of consultations, median (IQR)	2 (1–3)	3 (2–5)	
Wait-and-see/ watchful waiting, n (%)	34 (18.4)	78 (24.5)	6.1% (-0.02–0.14)
Advice, n (%)	45 (24.3)	102 (32.1)	7.8% (-0.01–0.16)
Medication prescription or advice, n (%)	68 (36.6)	175 (55.0)	**18.4% (0.09–0.28)**
Intra-articular injection, n (%)	17 (9.2)	40 (12.6)	3.4% (-0.03–0.09)
Aids and devices, n (%)	10 (5.4)	19 (6.0)	0.6% (-0.04–0.05)
Recommendation of/referral to other primary care practitioners, n (%)	37 (20.0)	147 (46.2)	**26.2% (0.08–0.25)**
Referral to secondary care, n (%)	137 (74.1)	174 (54.7)	**-19.4% (-0.37 - -0.19)**

Note: 95% CI = 95% confidence interval; † Patients without X-ray referral versus with X-ray referral, assessed using the Chi-squared test with Yates’s continuity correction; Bold values are statistically significant at 5% level

### Recorded management associated with type of knee OA diagnosis: narrative versus codified

Patients who were identified with an narrative diagnosis on the index date (n = 300) were significantly more likely to be referred for an X-ray and less likely to receive an intra-articular injection compared to patients identified with a codified diagnosis on the index date (n = 203) (absolute difference = 12.6% and − 8.3%, respectively) (Table 3). Among patients identified with a narrative diagnosis, 28.7% (n = 86) had a codified diagnosis within three years after the narrative diagnosis. In 75.6% (n = 65) of those patients, an X-ray referral was given to the patient before documenting knee OA with a code (Table 4).

**Table 3:** Differences in management between patients with codified diagnosed knee OA versus narratively diagnosed knee OA on index date

	Codified diagnosed patients (n = 203)	Narratively diagnosed patients (n = 300)	Absolute difference (%) (95%CI) †
Total number of consultations, median (IQR)	3 (2– 5)	3 (2–4)	
Wait-and-see/ watchful waiting, n (%)	42 (20.7)	27 (23.3)	2.6% (-0.10–0.05)
Advice, n (%)	64 (31.5)	83 (27.7)	-3.8% (-0.04–0.12)
Medication prescription or advice, n (%)	100 (49.3)	143 (47.7)	-1.6% (-0.08–0.10)
Intra-articular injection, n (%)	33 (16.3)	24 (8.0)	**-8.3% (-0.15 - -0.02)**
Aids and devices, n (%)	16 (7.9)	13 (4.3)	-3.6% (-0.08–0.01)
Diagnostic work-up, n (%)	124 (61.1)	221 (73.7)	**12.6% (0.21–0.04)**
Recommendation of/referral to other primary care practitioners, n (%)	58 (28.6)	95 (31.7)	3.1% (-0.05–0.12)
Referral to secondary care, n (%)	111 (54.7)	173 (57.7)	3.0% (-0.06–0.12)

Note: 95% CI = 95% confidence interval; † Codified diagnosed patients versus narratively diagnosed patients, assessed using the Chi-squared test with Yates’s continuity correction; Bold values are statistically significant at 5% level

**Table 4:** Management before and after codified diagnosis among patients identified with a narrative diagnosis on the index date with codified diagnosis later in time (n = 86 patients)

	Period before first codified diagnosis	Period after first codified diagnosis
Total number of consultations, median (IQR)	2 (1– 3)	1 (0–3)
Wait-and-see/ watchful waiting, n (%)	11 (12.8)	10 (11.6)
Advice, n (%)	16 (18.6)	14 (16.3)
Medication prescription or advice, n (%)	32 (37.2)	33 (38.4)
Intra-articular injection, n (%)	3 (3.5)	10 (11.6)
Diagnostic work-up, n (%)	65 (75.6)	12 (14.0)
Aids and devices, n (%)	4 (4.7)	0 (0.0)
Recommendation of/referral to other primary care practitioners, n (%)	16 (18.6)	17 (19.8)
Referral to secondary care, n (%)	36 (41.9)	30 (34.9)

## Discussion

This study determined patterns of knee OA management as recorded by GPs in EHRs from general practices. An X-ray referral was the most widely recorded management modality. The next most widely recorded management modalities were a referral to secondary care and medication prescription or advice. Records of recommendation of/referral to other primary care practitioners were found in only one third of the patients. Advice to lose weight was least common. Records of medication prescriptions or recommendation of/referral to other primary care practitioners were found more frequently in patients with an X-ray referral compared to patients without, while records of secondary care referrals were found less frequently. Records of an X-ray referral were often found in narratively diagnosed knee OA patients before GPs recorded a code for knee OA in their EHR.

Current guidelines [6–10] recommend a stepped-care approach for knee OA care where management should start with key management modalities and more intensive or invasive management modalities should only be used in a later stage of the disease. Key modalities include education, exercise and weight management, but previous studies showed that use of such modalities remains low [12–16, 28–34]. Similarly, our results showed that GPs report having given advice on education and self-management in only 17% of the knee OA patients, advice on exercise/physical activity/sports in 14% and advice on weight loss in 1%. Also, the percentage of patients with records of a recommendation of/referral to other primary care practitioners or advice on exercise/physical activity/sports during the first GP consultation was low, while the percentage of patients with a record of a secondary care referral for more invasive management modalities during the first GP consultation was high. This might imply an underutilization of a stepped-care approach for knee OA and can lead to low quality of care, redundant healthcare consumption, high healthcare costs, poor healthcare outcomes, and low patient and healthcare provider satisfaction [30, 31].

Similar to a previous study [18], our results show a wide gap between what guidelines recommend regarding X-rays and what GPs record doing for knee OA patients. Current guidelines [6–10] do not recommend the use of X-rays for diagnosing knee OA, but our results showed that a record of an X-ray referral at the first consultation is common. The use of X-rays is associated with unnecessary healthcare costs and can lead to the wrong impression among patients that OA is caused by ‘wear and tear’ with damage visible on X-rays [17]. A previous qualitative study showed that GPs often request X-rays for OA to feel more confident about their diagnosis [35]. This may also explain the high percentage of patients with a record of referral to secondary care at the first GP consultation in the current study. In addition, our study found that patients whose GP had requested an X-ray were more likely to have a record of medication prescription or advice and referral to a physiotherapist, and less likely to have a record of a referral to secondary care. This could indicate that GPs also use X-ray results to convince patients that invasive management modalities for knee OA (e.g. surgical treatment requiring a GP referral to secondary care) are not yet indicated. On the other hand, GPs’ reliance on X-rays for diagnosing knee OA may also lead to patients being incorrectly labelled as not having knee OA, since previous studies have shown that clinical knee OA does not always overlap with radiographic knee OA (and vice versa) [36, 37]. Another remarkable finding in the current study was that in narratively diagnosed patients, records of an X-ray referral were often found before GPs recorded a code for knee OA in their EHR. This suggests that GPs may be more confident about recording knee OA with a code when they have the support of the results of an X-ray.

A strength of the current study is the use of the IPCI database, which contains real-world data and covers a representative sample of the Dutch population [22, 23]. In addition, previous research on OA using EHR-based data focused on codified data alone which is likely an overrepresentation of more severe OA patient [38, 39]. Also, the IPCI database contains codes for medication prescription, but over-the-counter medicines (e.g. paracetamol) are not always reported because they are often advised instead of prescribed by GPs. In the current study, we collected information on GPs’ management from codified data and free text, and therefore also included over-the-counter medication advised by the GP rather than only medication prescribed and codified in EHRs.

A limitation of this study is that findings from the current study were based on data collected from information recorded by GPs in EHRs, which may not fully reflect the actual healthcare provided by GPs during the consultations. This could be true especially for advice provided during consultation but not registered in the EHR. We are not aware of previous studies evaluating the differences between EHR-documented care and actual care provided to patients with OA in general practice. Thus, the impact of this limitation on the findings of this study remains unclear. Also, the patient’s own actions to control their symptoms are not included in EHR-based data, such as consulting a physiotherapist on their own initiative. Patients in the Netherlands have been able to access physiotherapy care without a GP referral since 2006 [40]. Our study may underestimate the utilization of conservative management as it does not capture physiotherapy care without a GP referral. Linking routine healthcare data from general practices and physiotherapy is recommended for future studies to longitudinally assess the conservative management approach for knee OA across the borders of GP care. In addition, our study did not explore the impact of changes in healthcare policies and patient characteristics on the patterns in the management of knee OA by GPs. To gain deeper insights into the implications of the findings of this study, future research in this direction is warranted.

There are three main implications of the findings from the current study. First, results indicate that there is a great need for a better implementation of non-surgical management of knee OA in general practice. Strategies to facilitate this should focus on the first presentation of the patient with knee complaints in general practice with more focus on advice on for example self-management strategies, as the current study shows indications for underutilization of the stepped care which often starts at the first GP consultation. Second, this study demonstrated the overuse of X-rays in general practice for diagnosing knee OA. Helping GPs to perform more extensive history taking and physical examination in patients with knee complaints may give GPs more confidence in their ability to reach a diagnosis without the need for an X-ray. Furthermore, studies [41, 42] have shown that the patients’ preferences and beliefs may influence GPs’ decision to refer patients for diagnostic imaging, as GPs seem to be uncomfortable with rejecting patients’ requests for X-rays. Patient education about the role of diagnostic imaging for the management of knee OA may also help reduce X-rays in the general practice setting. Third, although this study showed that the type of documentation (narrative or codified) of knee OA in EHRs does not have consequences for clinical practice, it is still important for GPs to improve the quality of the codified recording for adequate reuse of routine healthcare data for research purposes.

## Conclusions

This study determined patterns of knee OA management recorded by GPs in a real-world setting using EHR-based data from general practices. An X-ray referral was the most widely recorded management modality, most often at the initial consultation. Also, this study showed indications for the underutilization of a stepped-care approach for knee OA,. These findings emphasize the importance of a better implementation of non-surgical management modalities of knee OA in general practice, especially during the first GP consultation, and on initiatives for reducing the overuse of X-rays for diagnosing knee OA in general practice.

### Electronic supplementary material

Below is the link to the electronic supplementary material.

Supplementary Material 1

## Data Availability

The aggregated data are available on request from the corresponding author.
