# Supplementary material for: Patterns of knee osteoarthritis management in general practice: a retrospective cohort study using electronic health records
Source: BMC Prim Care. 2024 Jan 2;25:2. doi: 10.1186/s12875-023-02198-z (PMC10759465; doi:10.1186/s12875-023-02198-z)
Supplement: Supplementary file 1 — Supplementary Material 1 [file 12875_2023_2198_MOESM1_ESM.docx]

**SUPPLEMENTARY FILES**

**Supplementary File 1. Details of the study design**

**
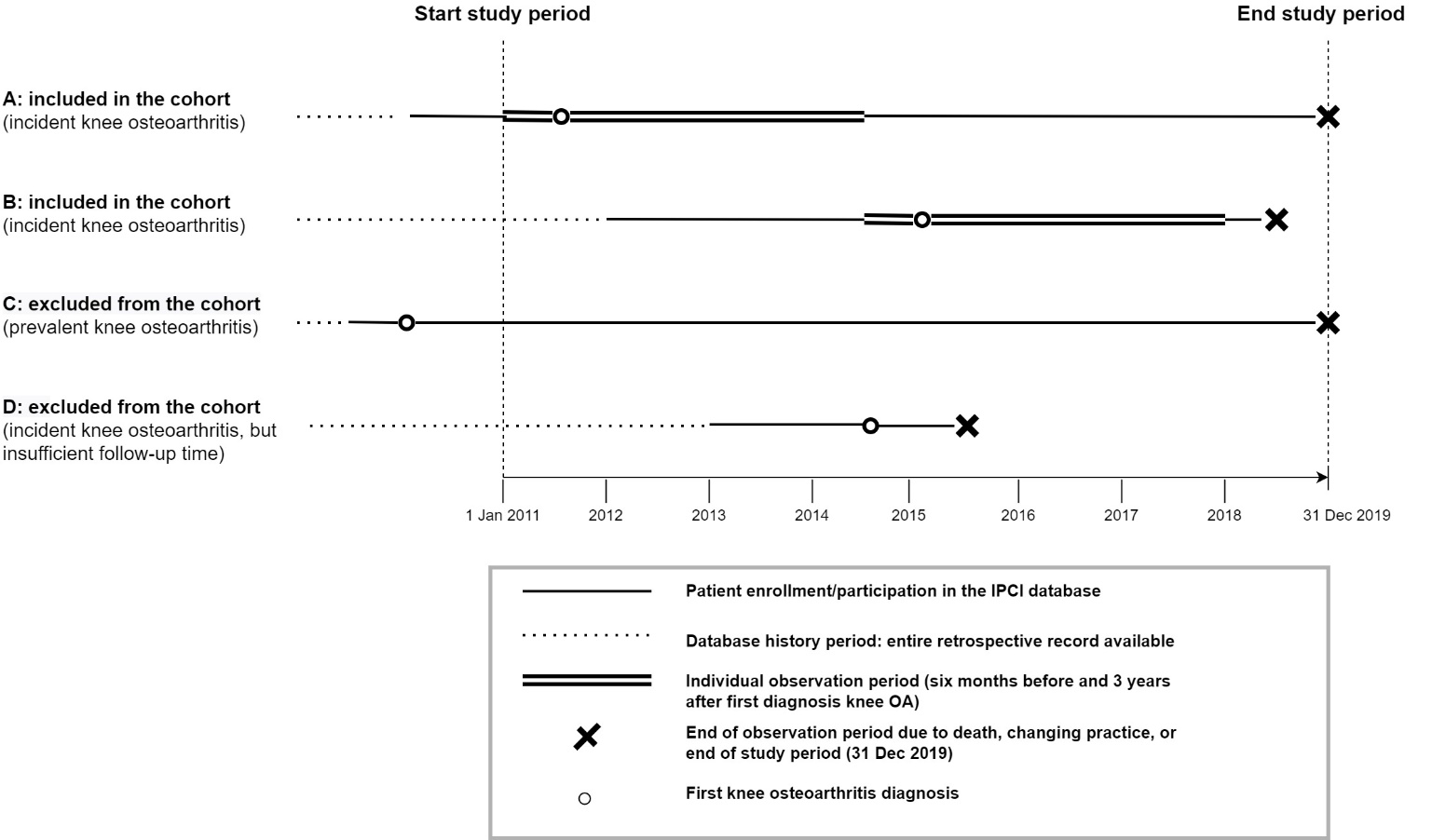
**

Figure 1 shows four examples of patients in the study cohort (A-D). The timeline shows how the study cohort was defined. The study period started on 1 January 2011 and ended on 31 December 2019. Patients with a first knee OA diagnosis (i.e. incident event) between July 2011 and December 2016 were included in the cohort (Patients A and B). A first knee OA diagnosis was defined as an incident if the first diagnosis was given during the study period (between 1 July 2011 and 31 December 2016) (patients A and B). These patients needed to have valid database information available for at least six months before and three years after the knee OA diagnosis, since patients in the study cohort were followed from six months before until three years after the first diagnosis of knee OA (Patients A and B). The entire set of retrospective records available for patients was used to exclude prior knee osteoarthritis when defining an ‘incident knee OA diagnosis’ (patient C). The IPCI database is an open cohort, meaning that patients can also enter the database after the start of the study period (1 January 2011) and stop before the end of the study period (31 December 2019) due to death or because they changed practice (Patient B). Patients were excluded from the cohort for the present study in case of death or changing practice within three years after the first knee OA diagnosis, since these patients do not have information available for the total follow-up period of three years (Patient D).

**Supplementary File 2. ICPC codes for comorbidities**

| Comorbidity | ICPC codes |
| --- | --- |
| Hypertension | K86 Essential hypertension without organ damage  K87 Hypertension with organ damage / secondary hypertension  F83.02 Hypertensive retinopathy |
| Hyperlipidaemia | T93 Lipid Metabolism Disorders  • T93.01 Hypercholesterolemia  • T93.02 Hypertriglyceridemia  • T93.03 Mixed hyperlipidaemia  • T93.04 Familial hypercholesterolemia/hyperlipidemia |
| Overweight | T82 Adiposity  T83 Obesity |
| Diabetes mellitus | T90 Diabetes mellitus |
| Myocardial infarction/ angina pectoris | K74 Angina pectoris  K75 Acute myocardial infarction  K76 Other / chronic ischemic heart disease |
| Transient ischaemic attacks/ stroke | K89 Transient ischaemic attacks  K90 Cerebrovascular accident |
| Peripheral arterial disease | K91 Atherosclerosis  K92.01 Intermittent claudication |
| Chronic Obstructive Pulmonary Disease | R95 Chronic Obstructive Pulmonary Disease |
| Asthma | R96 Asthma |
| Hip osteoarthritis | L89 Hip osteoarthritis |
| Spinal osteoarthritis | L84 Spinal osteoarthritis/spondylosis |
| Trauma | **ICPC codes** |
| Lower limb trauma | A80 Trauma/injury (general) |
|  | A81 Multiple trauma/internal injuries |
|  | L73 Fracture tibia/fibula |
|  | L74.02 Fracture ossa phalanges foot |
|  | L75 Fracture of the femoral column |
|  | L76.07 Other fracture – Fracture pelvis |
|  | L76.08 Other fracture – Fracture patella |
|  | L78 Sprain/ strain knee |

**Supplementary File 3. Information extracted from electronic health records of included patients per category**

| Overarching category | Detailed information extracted from EHRs |
| --- | --- |
| Wait-and-see/ watchful waiting | Wait-and-see/watchful waiting |
| Recommending a follow-up consultation | Advice on follow-up consultations |
| Education and self-management | Lifestyle factors |
|  | Treatment options for OA |
|  | X-ray has no added value |
|  | Inform and advice on disease OA |
|  | Applying ice/cold compress |
|  | Elevation (raising the knee above the level of the heart) |
|  | "Provision of information" (not documented what type of information) |
|  | "Advice" (not documented what type of advice) |
|  | "Instruction" (not documented what type of instruction) |
| Advice to lose weight | Advice to lose weight |
| Advice on exercise/physical activity/sports | Physical activity |
|  | Sports |
|  | Adjust joint load on knee pain |
|  | Balance between load and load capacity of the joint |
|  | Exercise |
| Advice to take rest or exercise less/reduce physical activity | Exercise less/reduce physical activity |
|  | Take rest |
| Any oral or topical medication prescription or advice | Paracetamol |
|  | Paracetamol codeine |
|  | Oral NSAID |
|  | Topical NSAID |
|  | Paracetamol combined with NSAID |
|  | Opioid |
|  | Opioid combined with paracetamol (Zaldiar) |
|  | Glucosamine |
|  | General advice on medication (not specified in EHR) |
| Intra-articular injection | Intra-articular injection administered by GP |
| Aids and devices | Walking aids (e.g. crutches) |
|  | Compression stocking |
|  | Footwear/insoles |
|  | Taping/bracing/bandage |
| Diagnostic work-up | X-ray |
|  | MRI |
|  | Ultrasound |
|  | Laboratory blood tests |
|  | Not clearly documented |
| Recommendation of/referral to other primary care practitioners | Physiotherapist |
|  | Dietician |
|  | Mensendieck |
|  | Podiatrist |
| Referral/advice to secondary care | Orthopaedic surgeon |
|  | Rheumatologist |
| Surgical intervention | Arthroscopy |
|  | Joint replacement (either total or hemi) |
|  | Distraction (therapy) |
|  | Osteotomy |
|  | Arthrodesis |

**Supplementary File 4. Management by the GP before and after knee OA diagnosis**

|  | 6 months before index date | From index date up to 3 years | Total observation period (6 months before and 3 years after index date) |
| --- | --- | --- | --- |
| Total number of GP consultations for knee OA, median (IQR) | 1 (0, 1) | 3 (1, 3) | 3 (2, 4) |
| Wait-and-see/ watchful waiting, n (%) | 37 (7.6) | 83 (16.5) | 112 (22.3) |
| Advice, n (%)   - Recommending follow-up consultation, n (%) - Education and self-management, n (%) - Advice to lose weight, n (%) - Advice on exercise/physical activity/sports, n (%) - Advice to reduce physical activity/exercise or to take rest, n (%) | 38 (7.6)  3 (0.6)  20 (4.0)  2 (0.4)  13 (2.6)  9 (1.8) | 121 (24.1)  1 (0.2)  71 (14.1)  5 (1.0)  61 (12.1)  17 (3.4) | 147 (29.2)  4 (0.8)  85 (16.9)  6 (1.2)  71 (14.1)  25 (5.0) |
| Medication prescription or advice, n (%)   - Paracetamol, n (%) - Oral NSAID, n (%) - Topical NSAID, n (%) - Paracetamol combined with NSAID, n (%) - Opioid, n (%) - Opioid combined with paracetamol (Zaldiar) , n (%) - Glucosamine, n (%) - General advice on medication, n (%) | 90 (17.9)  29 (5.8)  56 (11.1)  5 (1.0)  3 (0.6)  10 (2.0)  2 (0.4)  1 (0.2)  0 (0) | 196 (39.0)  81 (16.1)  114 (22.7)  18 (3.6)  10 (2.0)  31 (6.2)  12 (2.4)  0 (0)  4 (0.8) | 243 (48.3)  105 (20.9)  150 (29.8)  23 (4.6)  13 (2.6)  37 (7.4)  13 (2.6)  1 (0.2)  4 (0.8) |
| Intra-articular injection, n (%) | 4 (0.8) | 55 (10.9) | 57 (11.3) |
| Aids and devices, n (%) | 11 (2.2) | 22 (4.4) | 28 (5.6) |
| Diagnostic work-up, n (%)   - X-ray, n (%) - Ultrasound, n (%) - MRI, n (%) - laboratory blood test, n (%) | 202 (40.2)  178 (35.4)  9 (1.8)  24 (4.8)  8 (1.6) | 173 (34.4)  153 (30.4)  10 (2.0)  21 (4.2)  8 (1.6) | 345 (68.6)  318 (63.2)  19 (3.8)  44 (8.7)  16 (3.2) |
| Recommendation of/referral to other primary care practitioner, n (%)   - Physiotherapist, n (%) - Other practitioner (podiatrists, Mensendieck or dietician), n (%) | 39 (7.8)  36 (7.2)  4 (0.8) | 127 (25.2)  125 (24.9)  4 (0.8) | 153 (30.4)  150 (29.8)  8 (1.8) |
| Referral to secondary care, n (%)   - Orthopaedic surgeon, n (%) - Rheumatologist, n (%) | 122 (24.3)  120 (23.9)  2 (0.4) | 202 (40.2)  200 (39.8)  2 (0.4) | 284 (56.5)  282 (56.1)  4 (0.8) |

**Supplementary File 5. Management by the GP before and after an X-ray (n=318 knee OA patients)**

|  | Period before X-ray request | Period from and after X-ray request |
| --- | --- | --- |
| Total number of consultations, median (IQR) | 0 (0 – 1) | 3 (2 – 4) |
| Wait-and-see/ watchful waiting, n (%) | 28 (8.8) | 56 (17.6) |
| Advice, n (%) | 25 (7.9) | 84 (26.4) |
| Medication prescription or advice, n (%) | 50 (15.7) | 150 (47.2) |
| Intra-articular injection, n (%) | 5 (1.6) | 38 (11.9) |
| Aids and devices, n (%) | 9 (2.8) | 13 (4.1) |
| Referral/advice to paramedics, n (%) | 22 (6.9) | 103 (32.4) |
| Referral to secondary care, n (%) | 9 (2.8) | 142 (44.7) |
